# Items

**Published:** 1878-12

**Authors:** 


### Items.

American Science.—There are two names in the extended and honored list of American *savants* which every student regards with the highest respect and pride; we allude to Professor Gray, of Cambridge, and Professor Dana, of Yale. These two investigators in different fields have done more to make American science respected the world over than any others; and they may be justly regarded as the highest living authorities in the departments of science in which they have labored. The great works to which they have devoted their lives are standard and authoritative among educated men everywhere, and will continue to be long after their authors have passed away. The "Mineralogy" of Dana, and Gray's "Botany," form as thorough and exhaustive compendiums of two departments of science as the present stage of learning affords. They are vast storehouses in which are hoarded the ripest scholarship and the results of the widest researches of the age.

These two distinguished men are now in the evening of life, but still hard at work. Every hour of every day finds them either in their cabinets among their rare collections of specimens, in the field, or at their desks; and we can only hope that life and strength may be spared to them for many years, that they may make further and even richer contributions to human knowledge. Great learning and exhaustive intellectual labor, as far as our observation extends, do not produce unsocial habits of life, or pride of position, or arrogance; on the contrary, the truly great man in science or literature is in a marked degree genial and condescending. The men under consideration are in social life simple, modest, kind, generous, approachable. Such noble natures have no place for the pedantries and follies which environ little minds, when fortuitous circumstances bring them into notice. The earnest seeker after knowledge, however humble or untaught, never meets with rude rebuffs from the truly learned; they are the helpers and patrons of the lowly, if capable and honest.

It is not unusual to honor in some public manner our distinguished poets and literary writers at certain important epochs in their lives, and we know the suggestion that in like manner we remember those who by their researches in science have shed such high honor upon the American name will be gladly received. It is true, no garlands we can weave will add to the lustre of the names of our great scientists, but some appropriate mark of public appreciation of their labors and acquirements it would be delightful to bestow,—a delight which might not be denied us by those so deserving of our homage and respect.—*Exch.*

A SECULAR view of the controversy between certain members of the Association of Superintendents of Insane Asylums and certain specialists in nervous diseases, is given in the following article from the *Utica Morning Herald:*

"Our news columns have contained mention of proceedings for libel against Dr. John P. Gray, of the State Lunatic Asylum, in behalf of Dr. W. A. Hammond, of New York. The case may command some attention, and deserves further notice. The basis of the suit is an address written by Dr. Eugene Grissom, superintendent of the Insane Asylum for North Carolina at Raleigh. This was printed in the *American Journal of Insanity*, published by the State Asylum, and of which Dr. Gray, as superintendent, is the editor. This address was printed as a paper read before the American Association of Medical Superintendents of the Insane, held in Washington last May. The paper was the subject of some discussion in that body, and was reported at some length in the Washington newspapers. Dr. Hammond made it the text of an open letter addressed to Dr. Grissom, in which he revels in vituperative language. In his address, Dr. Grissom does not mention the name of Dr. Hammond, but describes with sharp lines the "false expert," and cites certain passages in the works and career of Dr. Hammond in illustration. To Dr. Hammond's open letter, Dr. Grissom made reply in print, and applied his address with more directness to Dr. Hammond. Both gentlemen are pretty free of speech, and both express very moderate estimates of each other. Dr. Hammond, by rushing into print in response to Dr. Grissom's address, at first put his case before the tribunal of readers. It may be because he feels himself worsted there that he goes to the courts.

"So far as Dr. Gray is concerned, the verdict of all who will examine the facts must be that he has in no way transgressed the strictest code of etiquette, much less any statute. In accordance with uniform custom, there were accepted for publication in the *Journal of Insanity* the official proceedings of the American Association of Medical Superintendents of the Insane, and this address and the debate upon it were parts of that report. Such a document could hardly be refused, and any injury which Dr. Hammond has sustained must date back to the association. The position of Dr. Gray is different from that of editors in general. The *Journal of Insanity* is the property of the State, and his connection with it is solely as the servant of the State.

"Dr. Hammond seeks notoriety, and his purpose in this libel suit may be to secure attention where he has not been able to compel it otherwise. In view of his published response to the address of which he complains, no jury in the world would award him a copper of damages.

THE FOLLOWING CIRCULAR LETTERS will be read with interest:

WAR DEPARTMENT,

SURGEON GENERAL'S OFFICE,

*Washington, Sept.* 28, 1878.

GENERAL JOSEPH K. BARNES,
*Surgeon General, U. S. Army.*

GENERAL:—I have the honor to submit the following suggestions with regard to the approaching United States Census, and to request that if they shall seem to you worthy of attention, they may be brought to the notice of the Congressional Committees which have the subject under consideration.

Having been consulted on certain points relating to the mortality statistics of the last census, I have given special attention to their probable value as affording a means of judging of the condition of the public health, and of the prevalence of certain causes of disease in different localities, and as a result of this examination, it appears to me that interesting as they are, it would be possible to obtain data much more valuable, from both a scientific and economic point of view, in relation to the health of the people of the United States.

As is pointed out by the Royal Sanitary Commission of England, " however complete the registration of deaths may be, it cannot give a fair estimate of the sickness which is not fatal, it cannot indicate where or how these are to be prevented, it cannot tell the cost which is worth incurring for their diminution."

It is probable that results of the greatest interest and value might be obtained from a record of sickness, and especially of those forms which are known to be due to contagion or to special local conditions.

So far as I can learn, the only attempts to obtain a registration of disease in connection with a national census have been made in Ireland, where the disease with which each individual is suffering on the day of the count is recorded; and in Portugal, where a query is inserted as to sickness, but in what precise form I have not been able to ascertain.

As chairman of the Section of Hygiene and State Medicine of the American Medical Association, I have corresponded with a number of the members of the section, and with other skilled sanitarians, all of whom unite in the opinion that it is possible in the next census to obtain some data with regard to disease which will inaugurate a new branch of statistics in this country, and that it is highly desirable that the general government should take the lead in this matter.

The principal difficulty has been to obtain substantial agreement as to the questions upon which information is most desired, keeping in view the fact that these questions are to be asked and answered by unprofessional men.

As the result of the conferences above alluded to, and of careful study of what is practicable as well as what is desirable, I

venture to suggest the following five queries as being, if not the best, at least such as will bring out a vast amount of information which will be extremely interesting and useful to the political economist, the sanitarian and the physician.

The amount of information which moderately complete answers to them will give, is not to be estimated from the questions themselves only—for it should be remembered that of each individual to whom these queries apply, the age, sex, color, parentage and occupation are also recorded in the schedules now in use, and hence the influence of these conditions separate or combined upon the diseases to which my proposed queries relate can be made to appear in various ways.

The queries suggested are as follows:

1st. Number of days during past year in which the person was unable to follow his or her usual occupation on account of disease (D) or injuries (I). (Attendance at school considered as an occupation.)

2d. Is the person sick on the 30th day of June; if so, name disease or injury.

3d. Is the case being treated in hospital (H), by a physician from a dispensary or public charity (C), by a private physician (P) or without a physician (N).

4th. Has the person during the past year had any of the following diseases, viz: Small pox or varioloid (S P or V); scarlet fever (Sc); measles (Me); diphtheria (D); typhoid fever (T F); malarial fever (M F) (includes ague, bilious fever and remittent fever); yellow fever (Y F); acute lung diseases (L D) (includes lung fever, pheumonia and pleurisy); acute rheumatism (A R); cerebro-spinal-meningitis (C S M).

5th. What has been the cost to the person (or head of the family on his account) during the past year from sickness, in—

*a.* Loss of wages or salary?

*b.* Cost of medical attendance, medicines and nursing?

These queries for the most part are self-explanatory. The second question is that used in the Irish census, the name of the disease being entered in the persons' own words (in Ireland it is often entered in Irish) leaving it to an expert at the central office to classify it as best he can. The fifth query can in most cases be answered only approximately, but for the working classes, at all events, the answers will be near enough to the truth to be of value. Very respectfully, your obedient servant,

(Signed) J. S. BILLINGS,

*Surgeon, U. S. Army.*

WASHINGTON, D. C., October —, 1878.

It has become a custom that the chairman of the section of hygiene and state medicine of the American Medical Association shall propose subjects for the special attention of the section.

In accordance with this custom, I would respectfully suggest the following subjects for consideration at the next meeting, viz:

I. State and Municipal Organizations for Public Hygiene, and their relations to Practitioners of Medicine.

II. Vital Statistics; including Statistics of Disease.

III. The Causation and Prevention of Yellow Fever.

Under the first heading, papers are promised upon: (a) The proper organizations of boards of health. (b) The relations of the Massachusetts system of medical examiners to boards of health. (c) The relation of State control of medical education to boards of health. (d) The relations of the code of ethics to State and municipal sanitary organizations.

The address of the chairman of the section will relate mainly to the second heading, and in this connection your attention is respectfully invited to the inclosed proposal for obtaining some statistics of disease in the next census, and if you approve of it, it is hoped that you will use your influence in its favor with Congress.

Under the third heading, the lessons of the recent epidemic and methods of investigation of yellow fever, will be considered.

Your assistance in the work thus indicated is respectfully requested. Please notify me as to whether you will undertake to prepare a paper for the section, or to aid in the discussions, indicating also the subject which you have selected.

Very respectfully and truly yours,

J. S. BILLINGS,

*Surgeon U. S. Army, and Chairman of the Section of Hygiene, American Medical Association.*

Messrs. E. H. Sargent & Co., dispensing chemists of this city, have republished, at their own expense, the valuable pamphlet on Metric Weights and Measures for Medical and Pharmacal Purposes, originally prepared for the U. S. Marine Hospital service, by Oscar Oldberg, Phar. D. It is intended to furnish a copy of this pamphlet to all reputable physicians in Chicago who desire to obtain it.

In this commendable enterprise Messrs. E. H. Sargent & Co. have taken a step which not only will greatly aid in extending a knowledge of the metric system, but which reflects great credit upon themselves. The Journal and Examiner takes pleasure in extending to them its congratulations.

Politics and Science.—Dr. A. W. Heise, who has held the office of physician to the Illinois State Penitentiary at Joliet for the past three years, was lately deposed from office "by reason of expiration of term of service."

Dr. Heise managed the medical affairs of the penitentiary with credit to the institution and himself. He instituted sanitary regulations which practically abolished endemic and epidemic disease within the prison. The ratio of sick in hospital is said to be lower than that of any other prison in the United States, as shown by his annual report, there being an average of six hospital cases from an average number of convicts amounting to over 1,700.

The commissioners have appointed a "homœopathist" to take the place of the prison physician.

Doubtful Novelties.—Dr. W. P. Gibbons, of California, does a good work in the October number of the Pacific *Medical and Surgical Journal*, by exposing the pretended new remedies from California called *Yerba Santa*, *Berberis Aquifolium*, *Cascara Sagrado* and *Yerba Reuma*. They have been pushed on the profession by a Dr. I. H. Bundy, and by the efforts of Parke, Davis & Co., of Detroit. We regret to add that several medical editors have either been entrapped or have deliberately been bargained into giving their sanction and aid to this form of mercantile exploitation of the profession and their patients.—*Med. & Surg. Rep.*

